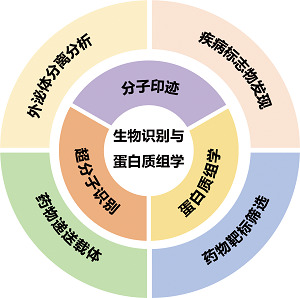# 单细胞与单颗粒外泌体分离分析进展与展望

**DOI:** 10.3724/SP.J.1123.2024.11001

**Published:** 2025-05-08

**Authors:** Aixiang BU, Guangyao WU, Lianghai HU

**Affiliations:** 吉林大学超分子化学生物学研究中心, 超分子结构与材料全国重点实验室, 生命科学学院, 吉林 长春 130023; Center for Supramolecular Chemical Biology, State Key Laboratory of Supramolecular Structure and Materials, Jilin University, Changchun 130023, China

**Keywords:** 单细胞外泌体, 单颗粒外泌体, 分离与分析, 微流控技术, 综述, single-cell exosomes, single-particle exosomes, separation and analysis, microfluidics, review

## Abstract

外泌体是细胞分泌的一种纳米级囊泡颗粒,由脂质双分子层包裹,它们在细胞通讯中发挥着重要的作用,并参与到多种生理及病理过程中,如免疫调节、血管生成以及肿瘤的发生与转移等。外泌体携带了来自母体细胞的多种生物分子,因此成为疾病标志物发现的一个重要载体。由于来自不同细胞亚群的外泌体具有显著的异质性,利用传统检测方法仅能获取样本中群体细胞的平均信息,而无法建立外泌体生物功能与亚型之间的明确关联。因此,为了深入探究外泌体的特异性并清晰区分不同亚型外泌体的特征,有必要在单细胞及单颗粒水平上对外泌体进行表征。常用的单颗粒外泌体表征技术主要包括流式细胞术、超分辨显微镜、原子力显微镜、表面增强拉曼光谱、邻近编码技术和质谱技术等。本文总结了基于微流控技术的单细胞外泌体分离分析方法及单颗粒外泌体表征技术的最新进展,并对新兴技术(如Olink蛋白质组学、点击化学和分子印迹技术等)在单细胞及单颗粒外泌体研究中的应用前景进行了展望。

## Abstract (en)

Exosomes are nanoscale vesicles secreted by cells and are encapsulated in lipid bilayers. They play crucial roles in cell communication and are involved in a variety of physiological and pathological processes, including immune regulation, angiogenesis, and tumor initiation and metastasis. Exosomes carry a variety of biomolecules from maternal cells and are therefore important vehicles for discovering disease markers. Traditional detection methods only provide average cell-population information for a given sample and cannot establish clear relationships between the biological functions of exosomes and subtype owing to the significant heterogeneity associated with exosomes from different cell subsets. Therefore, characterizing exosomes at the single-cell and single-particle levels requires exosome specificities to be further explored and the characteristics of various exosome subtypes to be distinguished. Commonly used single-particle exosome characterization technologies include flow cytometry, super-resolution microscopy, atomic force microscopy, surface-enhanced Raman spectroscopy, proximity barcoding assay and MS. In this paper, we summarize recent advances in the separation and characterization of single-cell exosomes based on microfluidics and provide future applications prospects for emerging technologies (such as Olink proteomics, click chemistry, and molecular imprinting) for studying single-cell and single-particle exosomes.

外泌体是细胞通过内吞途径产生的纳米级(40~160 nm)囊泡,它们具有磷脂双分子层结构,并且几乎存在于人体的所有体液中。^[1,2]^。外泌体的形成过程涉及早期核内体(EEs)、晚期核内体和多泡体(MVBs)^[3]^。外泌体富含多种生物分子,包括蛋白质、核酸、脂质和代谢物等,其在细胞间的通讯过程中起着重要的作用^[4]^。外泌体介导的细胞间通讯在调节细胞迁移、免疫反应、组织再生和肿瘤发展等细胞过程中至关重要^[5,6]^。外泌体膜上携带了由亲代细胞分泌的表面蛋白,这些蛋白是疾病诊断标志物的重要来源^[7,8]^。然而,外泌体具有粒径小、有效载荷低及异质性高等特点,它们在生物诊断领域的应用面临着巨大挑战^[9]^。体液中的外泌体来自不同细胞类型的多种亚群,这些外泌体在不同疾病类型以及不同患者个体之间展现出了显著的差异性^[10]^。利用传统的检测方法(如蛋白质印迹(Western blotting)^[11]^、酶联免疫吸附测定(ELISA)^[12]^和聚合酶链式反应(PCR)^[13]^)仅能获取样本中群体细胞的平均信息,而无法建立外泌体生物功能与亚型之间的明确关联。因此,有必要在单细胞和单颗粒水平上对外泌体进行研究,以获得精确的信息^[14]^。本文综述了基于微流控技术的单细胞外泌体分离分析方法及单颗粒外泌体表征技术的最新进展,并对具有潜力的新兴技术进行了展望,旨在为单细胞与单颗粒外泌体的研究提供有价值的参考。

## 1 基于微流控技术的单细胞外泌体分析平台

### 1.1 单细胞的获取与培养

获取和培养单细胞是实现单细胞外泌体研究的先决条件。基于微流控技术的单细胞分离和培养在单细胞外泌体研究中起着至关重要的作用^[15,16]^。微流控技术不仅能够从微观样品中分离出单个细胞,还可以更直观、更精确地评估细胞和外泌体的特性。单细胞外泌体的样本量很小^[17]^,而微流控装置可以集成多个腔室,对样品和试剂的需求量很小,十分契合这一需求。此外,微流控装置还适用于高通量的单细胞外泌体分析。通过将多个阀门集成到设备中,微流控技术能够同时调控多个单细胞的流动、分选和捕获过程,这不仅降低了人工成本,还最大限度地减少了样品污染的风险^[18]^。此外,微流控技术还可以与实时检测技术相结合,利用实时检测技术提供的记录来监测并分析单细胞外泌体的特征^[19,20]^。常见的单细胞外泌体微流控分离技术包括液滴微流控技术、基于陷阱的微流控技术和基于阀的微流控技术。

#### 1.1.1 液滴微流控技术

液滴微流控技术是一种将反应限制在nL级或pL级体积内的技术,可用于单细胞的获取、培养及各种下游分析^[21]^。液滴微流控的生成技术主要分为被动封装和主动封装。在被动封装过程中,液滴的形成主要依赖于流动剪切力与表面张力之间的相互作用,这一过程将连续流体分割成nL级的小而离散的液滴。通过调控流体流速、黏度和微通道几何形状等参数,可以实现对微通道内流体的精确控制,从而快速产生尺寸均一的液滴^[22]^。被动封装具有设备简单、操作简便的优点,然而,由于封装过程的随机性,含有单个细胞的微滴占比相对较低。为了提高封装效率,可将声力、电力、磁力或光学手段与被动封装相结合,以此实现具有可控性的主动封装。Lagerman等^[23]^将流动聚焦技术与超声振动结合,成功实现了尺寸可调的单细胞封装液滴的高通量生产。在该平台运行过程中,通过超声波能量控制液滴的断裂点,进而调节液滴的大小。Jiang等^[24]^开发了一种完全集成的纸基液滴微流控平台,该平台通过向分散于载体油中的单个液滴施加电场,实现了对酵母细胞的封装。Navi等^[25]^通过将磁场与微流控技术相结合,成功制备了全部由单个乳腺癌细胞(MCF-7)组成的水包水液滴,这些特定液滴在收集到的所有液滴中占比达到了100%。该方法依赖永磁体运行,且磁力作用不会对液滴内细胞的活力产生影响。Hu等^[26]^开发了一种荧光激活液滴形成技术,该技术能够生成封装有单个宫颈癌细胞(HeLa)的微滴。通过操控两相流体动力闸阀的开启与关闭,生成含有单个细胞的液滴,且单细胞的捕获效率高达82.5%。尽管声力、电力、磁力及光学手段的结合能够有效控制液滴的形成过程,但这些技术的引入也增加了微流控器件的制造成本和复杂性。Si等^[27]^通过对微流控芯片进行设计,成功实现了细胞有序排列以及直接的单细胞封装。这一成果显著促进了间充质基质细胞的抗炎反应进程,同时增强了间充质基质细胞向周围环境或体内传递具有治疗作用物质的能力。Ahmadi等^[28]^报道了一种可用于发现单克隆抗体的自动化单细胞液滴-数字微流控平台,并利用该平台成功实现了胶质瘤细胞的识别。Wang等^[29]^利用基于微阀的按需滴注技术以及实时图像处理手段,实现了无标签方式下的单细胞封装,对HeLa细胞的封装效率高达94.9%。综上所述,液滴微流控技术已成为获取和培养单细胞的重要方法之一。

#### 1.1.2 基于陷阱的微流控技术

基于陷阱的单细胞微流控技术主要依赖于微流控芯片上设计的微型陷阱结构,以实现对单个细胞的捕获^[30]^。根据捕集过程中细胞与陷阱区域表面的接触情况,该技术大致可以分为接触捕集技术和非接触捕集技术^[31]^。接触捕集技术利用微加工技术在微流控芯片上构建出特定形状和尺寸的陷阱结构。当细胞悬浮液在微流控通道中流动时,细胞会在流体作用力以及陷阱结构的约束下,精准地落入陷阱内,进而被稳定捕获。这些陷阱的大小可以根据待研究细胞的直径进行调整,以实现高通量的细胞操作。U型结构的微流控芯片在捕获单细胞方面展现出了最高的效率,其不仅能有效捕获单细胞,还能对捕获的单细胞进行长期、连续的培养和观察,同时这也减少了样品和试剂的消耗^[32]^。Gao等^[33]^提出了一种可用于分析单细胞代谢物的微流控芯片(SingMAC),旨在模拟肿瘤外渗过程中单细胞微环境的形成。该SingMAC由密集的流体动力学钩状陷阱阵列组成,每个陷阱均有一个相邻的腔室。结果表明,99%的陷阱实现了单个细胞隔离,并能够在肿瘤外渗过程中成功捕获单个肿瘤细胞。Narayanamurthy等^[34]^开发了一种基于陷阱的微流控灌注平台,该平台能够装载、观察、处理及记录单个卵母细胞和胚胎,并在设备内部完成细胞孵育过程,从而减少了因细胞转移所导致的样品损失,为微流控技术在生物学领域的应用开辟了新的途径。Wang等^[35]^设计了一种双孔结构的阵列芯片,该芯片利用重力将细胞加载到双孔中,随后通过磷酸盐缓冲液将反应孔中多余的细胞排出。该芯片的单细胞捕获效率高达75.8%,并能够在单细胞水平上测定人慢性髓系白血病细胞(K562)内的*β*-半乳糖苷酶活性。Zhu等^[36]^利用光刻技术制备了一种高通量微孔阵列芯片,用于单细胞的捕获(图1)。在捕获过程中,细胞在重力作用下落入微孔中,且这些微孔中的细胞分布遵循泊松分布规律。介电泳(DEP)是一种非接触捕集技术,通过对细胞施加DEP力可以实现单细胞的捕获。Bai等^[37]^通过集成DEP技术,在实时成像显微镜下观察到单个小鼠胚胎成纤维细胞(NIH/3T3)的捕获率高达91.84%,突破了细胞捕获的泊松极限。在该平台中,流速、电压和电波等参数的调控对于实现细胞的高效分离至关重要。在基于陷阱的微流控技术研究中,芯片开发领域涌现出众多新尝试。Zhang等^[38]^将用于粒子处理和流体控制的数字掩模实时原位生成功能集成到基于数字微镜器件(DMD)的传统投影光刻技术中,发展了一种新的原位数字投影光刻技术。该技术可以在预设位置快速形成精确的陷阱或过滤器,以捕获目标颗粒,这为基于陷阱的微流控平台发展提供了新的思路。基于陷阱的微流控技术具有设计简单和易于操作等特点,在单细胞分离和培养方面展现出了巨大的潜力。

**图1:**
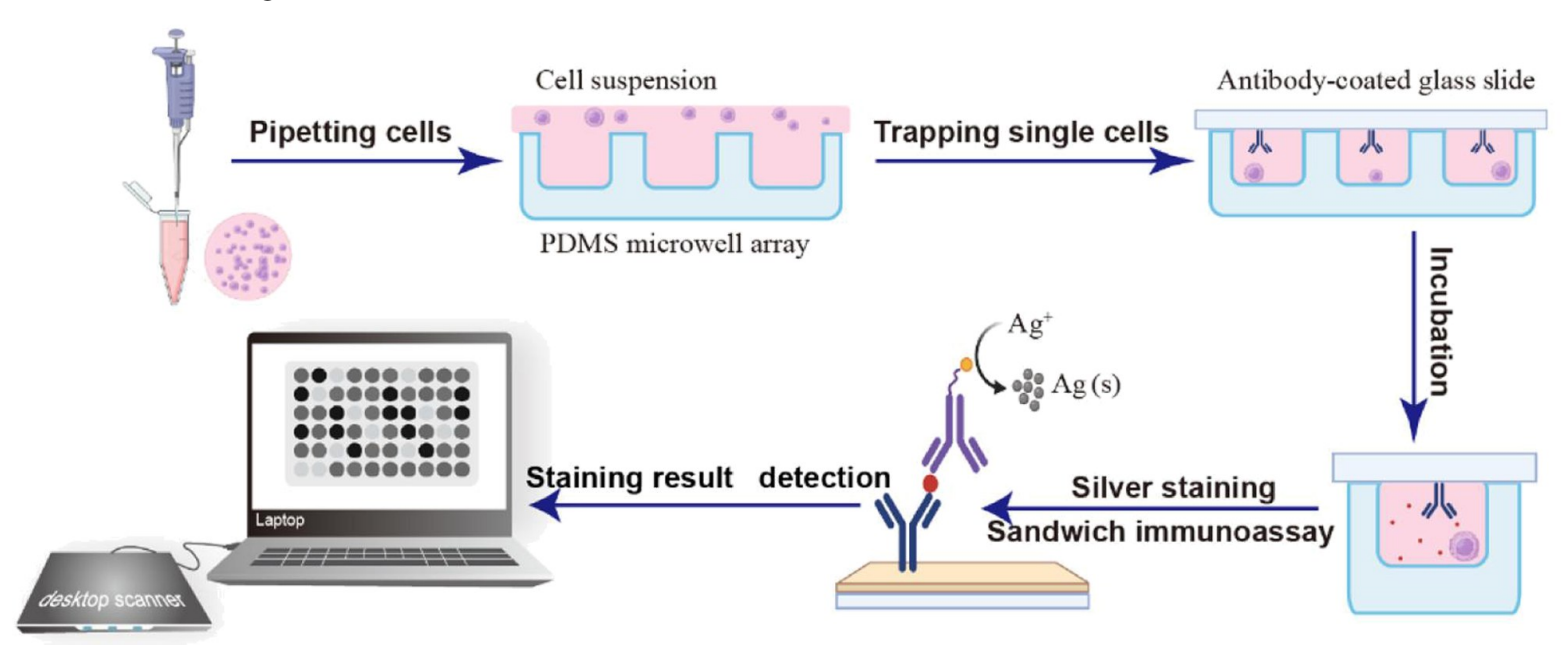
基于陷阱的高通量微孔阵列芯片对单细胞的捕获示意图^[36]^

#### 1.1.3 基于阀的微流控技术

基于阀的微流控装置可以通过阀门巧妙地将其划分多个独立隔间,每个隔间都能进行单独的反应。根据需要打开或关闭阀门,这些装置能够捕获和培养细胞。在该装置中,阀结构的设计以及流体的流量和黏度是实现细胞高效分离的关键因素。基于阀的微流控系统能够在芯片中实现自动化操作,极大地辅助研究人员完成单细胞分析所需的各种实验功能。因此,基于阀的微流控技术能够精确地模拟细胞微环境的动力学特性,展现出高精度和高度可控性^[39]^。Sun等^[40]^设计了一种微流控进料平台,该平台采用双层气动阀结构,能够同时在薄片上固定和处理细胞,并对处理后的细胞进行无损收集,以供后续分析。Briones等^[41]^通过探究几何形状、阀门尺寸与密封压力之间的关联性,对微流控平台进行了设计与优化。他们设计了一种微流控装置,该装置包含多达5000个流体动力陷阱及相应的微阀,便于实现高通量的单细胞蛋白分区表达。Moradi等^[42]^报道了一种可用于单细胞分析的微流控横杆设计,该设计利用了液滴条形码技术,通过增加横杆上的阀门数量,提高了对单侧故障的容错能力,同时保持了高效的分离性能。尽管基于阀的微流控技术能够解决操作性受限的问题,但其设备制造过程的复杂性和操作难度却增加了相应的成本。

以上3种基于微流控的单细胞分离技术,在细胞捕获和培养方面各具优缺点。液滴微流控技术具有简单、高效等特点,但所产生的微滴不适用于单细胞的长期培养。基于陷阱的微流控技术具有设计简单和高通量等特点,然而有时需转移培养才能成功获取单个细胞。基于阀的微流控技术具有高精度和高度可控性等优点,但其设计复杂且成本较高。目前,利用微流控技术对单细胞外泌体的研究比较有限,如何精确捕获并培养单细胞仍是需要探索的问题。

### 1.2 单细胞外泌体的分离方法

外泌体的分离是实现单细胞外泌体研究的关键步骤,其分离效率对后续研究的成败至关重要。目前,许多方法已应用于外泌体的分离,包括超离心法、沉淀法、尺寸排阻色谱法和反向富集法^[43,44]^,然而这些方法所需的样本量较大,不适用于单细胞水平的外泌体研究。相比之下,基于抗体的分离方法不受样本量的限制,是当前微流控技术中单细胞外泌体分离的主要手段^[45]^。外泌体跨膜蛋白(CD9、CD63、CD81)位于外泌体表面,其能够与固定在玻璃侧或微通道内的相应抗体结合,进而实现外泌体的特异性富集与分离。Son等^[46]^通过将抗CD63抗体修饰于微球表面,获得了具有特异性识别能力的抗CD63磁珠。将该磁珠与可重构的微流控装置结合,并对外泌体进行捕获,实现了单个肝癌细胞(HepG2)所释放外泌体的动态表征。Chiu等^[47]^将抗CD63磁珠固定于载玻片上,用以富集乳腺癌细胞(MCF-7和MDA-MB-231)分泌的外泌体。Cai等^[48]^基于抗体条形码的微芯片平台,对单细胞外泌体进行多重分析,为细胞间通讯和肿瘤微环境的研究提供了有力的工具。为了全面捕获外泌体,Ji等^[49]^将捕获抗体(如抗CD63、抗CD81、抗CD9)修饰在微阵列表面,从而在玻璃板上形成抗体条形码,进而应用于人口腔鳞状细胞癌(OSCC)细胞系的外泌体捕获和表征。Nicoloff等^[39]^设计了一种双层微流控装置,该装置配备有72个柱子和两个同心阀。通过在该装置表面固定抗CD81抗体,能够捕获由单个MCF-7细胞分泌的外泌体。

除了抗体之外,基于适配体的方法在单细胞外泌体分离方面也展现出巨大潜力。核酸适配体是一种短的单链寡核苷酸,其对特定靶标(如CD9、CD63、CD81)具有较高的亲和力和选择性^[50]^。与抗体相比,适配体具有制造成本低、易于修饰及洗脱条件相对温和等优点。将适配体修饰在微流控装置的表面,可用于单细胞外泌体的分离^[51]^。Zhou等^[52]^将适配体修饰在微流控装置的微通道内,通过靶向外泌体所携带的CD63和蛋白酪氨酸激酶-7(PTK7),实现对外泌体的高效分离。除了固定在微通道中,适配体还能够以自由形式与外泌体结合。Liu等^[53]^使用7种核酸适配体对外泌体表面蛋白进行互认,经荧光诱导剂修饰后,核酸适配体可对游离的血清外泌体进行标记,并可通过热富集和线性判别分析技术对这些外泌体进行检测和分类。Ren等^[54]^利用对CD63具有高亲和力的微流控芯片,实现了血清中外泌体的快速富集和灵敏检测。Zhu等^[55]^采用了一种双重标记策略,结合蛋白质特异性适配体标记和代谢聚糖标记,实现了外泌体蛋白糖基化的原位可视化及相关生物学功能研究。

除了基于外泌体表面标记物的分离方法外,还有若干种基于外泌体物理特性的分离方法,如微流控过滤和确定性横向位移(DLD)等。微流控过滤的工作原理类似于普通过滤,它依据外泌体的大小来实现分离^[56]^。同样地,DLD也是根据外泌体的直径来实现有效的分离。在特定的柱阵列微流控平台上,不同粒径的颗粒会从不同的出口流出;与直径较大的颗粒相比,小颗粒会发生横向位移^[57]^。此外,还有一些基于外力场的技术被应用于外泌体的分离,如声流技术和DEP分离。声流技术的分离原理是基于不同粒径的颗粒在声场环境中所受到的作用力不同,直径较大的颗粒受到的声场力更强,其在微流控平台上的偏转距离更大,而直径较小的颗粒的偏转距离则更小^[58]^。外泌体的DEP分离原理基于外泌体的介电性质,不同的外泌体在DEP场中将受到不同的电场力。最近,Feng等^[59,60]^将具有两亲性的两性霉素B(AMB)分子接枝到高度支化的树枝状聚合物上,制备了一种两亲性超分子探针(ADSP)。该探针可以高效地嵌入外泌体的磷脂双分子层膜,利用探针与外泌体外膜之间的多价相互作用,研究实现了外泌体的高效捕获(图2)。此外,将ADSP修饰在硝化纤维素(NC)膜上,能够一体化地完成外泌体的富集与蛋白质的原位检测。将该探针与微流控平台相结合,有望应用于单细胞水平的外泌体研究。

**图2:**
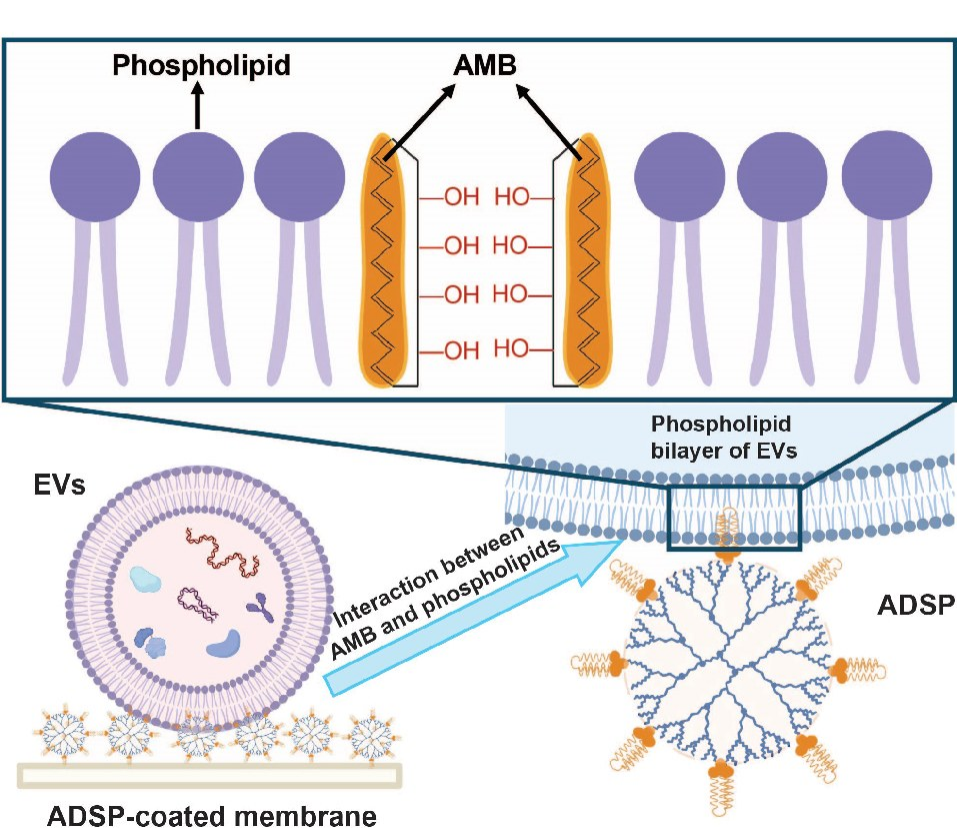
基于ADSP的外泌体分离示意图^[60]^

### 1.3 单细胞外泌体的分析与应用

对单细胞外泌体进行精确分析,有助于揭示不同单细胞外泌体在膜蛋白组成和含量上的异质性。目前,已有多种先进的检测工具(如荧光光谱、拉曼光谱、质谱(mass spectrometry, MS)和流式细胞术((flow cytometry, FCM))等)被应用于单细胞外泌体的分析中。Son等^[46]^将微流控装置与荧光显微镜结合,实现了对单个HepG2细胞分泌外泌体的动态检测与计数。利用微流控装置对多个单细胞同时进行多重分析,是达成单细胞高通量分析的关键前提。Ji等^[49]^首先采用光刻技术制备了条形模具,随后将聚二甲基硅氧烷(PDMS)预聚物倾注在该模具上,形成PDMS微芯片。研究共设计了两套微室阵列:一套用于细胞培养以分泌外泌体,另一套则用于涂覆捕获抗体。将捕获抗体涂覆在第二套微室阵列中,从而在玻璃板上构建出抗体条形码;接着,将该玻璃板置于微芯片顶部,共同孵育过夜以实现外泌体的捕获。捕获完成后,将特异性抗体CD63与外泌体结合;随后,加入链霉亲和素标记的藻蓝蛋白(APC)或藻红蛋白(PE)复合物,这些复合物能够与CD63抗体上的生物素特异性结合,构建成免疫三明治结构;由于APC和PE复合物携带荧光基团,使得外泌体被荧光标记,最后通过荧光扫描仪对玻片进行扫描检测,成功实现了对超过1000个单细胞来源外泌体的多重分析。

在此基础上,Zhu等^[36]^将免疫金银染色技术与泊松分布统计方法相结合,构建了一种便携式检测平台,可在桌面扫描仪上实现无需细胞计数的高通量单细胞外泌体分析。这一便携式检测平台被用于分析人口腔鳞癌细胞系以及口腔鳞癌患者原代细胞所分泌的不同表型的外泌体。Song等^[61]^利用一个集成了单细胞捕获芯片与空间编码外泌体抗体条形码芯片的微流控平台,成功鉴定了卵巢肿瘤细胞中独特表型外泌体热休克蛋白70(HSP70)和上皮细胞黏附分子(EPCAM)的特定功能细胞亚群。在生物学和临床研究中,该平台在探索单细胞生物标志物方面具有巨大的潜力。Nikoloff等^[39]^将微流控技术与全内反射荧光显微镜(TIRFM)结合,设计了一个双层微流控装置,该装置配备有PDMS柱和同心阀,可用于单细胞外泌体的鉴定。通过修饰在管腔表面的CD81抗体对单细胞外泌体进行捕获,并使用膜联蛋白A5(ANXA5)、肿瘤易感基因101蛋白(TSG101)、HSP70和CD63抗体对外泌体进行免疫染色,随后根据TIRFM观察到的外泌体表面标记蛋白的荧光信号来确定外泌体的位置。研究结果显示,结合免疫染色与四色TIRFM技术,能够区分出由单个细胞或多个细胞分泌的外泌体数量。Chiu等^[47]^运用微流控技术设计了一种PDMS网格装置,用于分析单细胞外泌体。该装置能够非侵入性地捕获单细胞释放的外泌体,并允许在不同时间点对这些外泌体进行研究。通过监测特定扰动条件下单个细胞释放的生物标志物信号,该装置能够进一步提供关于单细胞行为的定量信息。Zhang等^[62]^报道了一种双纳米孔生物传感器,该传感器基于DNA适配体与外泌体表面蛋白的直接结合,用于外泌体检测。Wang等^[63]^构建了一种基于微液滴-表面增强拉曼光谱(SERS)的单细胞检测平台,该平台可在单细胞水平上分析外泌体蛋白。将单细胞与捕获探针(即免疫磁珠(iMBs))及报告探针(iSERS tags)一同封装在微液滴内部。在微液滴环境中,可观察到一种独特的界面定向聚集(IOA)现象,即带负电的细胞膜倾向于吸引电中性的iMBs,使其自发地向细胞膜表面聚集;随后,外泌体蛋白与iSERS tags通过生物偶联作用结合到iMBs上,形成三明治夹心结构,通过采集iSERS tags的SERS信号,即可实现对外泌体蛋白的分析。鉴于单细胞外泌体的分子载荷量较低,对外泌体标志物的检测不应仅局限于蛋白质,信使RNA(mRNA)同样是一种有效的标志物。Zhou等^[64]^开发了一种高通量液体活检纳米芯片(HNCIB),该芯片能够在单细胞水平上同时检测外泌体的蛋白质、mRNA和微小RNA(miRNA),实现了对外泌体表面和腔内多种类型分子标志物的同时检测。此外,该技术对单细胞外泌体的检测时长仅为6 h,且样本需求较小(约为90 μL)。Nguyen等^[65]^设计了一种免疫金生物芯片(AuSERP)来对单个外泌体的mRNA和膜蛋白进行联合检测。结果表明,在癌症诊断和免疫治疗反应预测方面,单细胞外泌体mRNA优于单细胞外泌体蛋白。

## 2 单颗粒外泌体的表征技术

外泌体的物理特性,涵盖尺寸、形态、数量以及机械性能,构成了其研究的另一个核心领域。常用的单颗粒外泌体表征技术主要有流式细胞术、超分辨显微镜(super-resolution microscopy, SRM)、原子力显微镜(atomic force microscope, AFM)、SERS、质谱和邻近编码技术(proximity barcoding assay, PBA)等。

### 2.1 流式细胞术

流式细胞术是一种先进的光散射技术,能够快速定量分析流经聚焦光源悬浮液中的单个细胞或类细胞大小的颗粒^[66]^。然而,由于灵敏度的局限,传统FCM难以探测到尺寸小于300 nm的囊泡,这限制了其在表征生物标志物水平较低的单颗粒外泌体方面的应用。Shen等^[67]^将传统的FCM与靶启动工程(TIE)结合,在每个外泌体的DNA纳米结构上进行单颗粒外泌体的计数和表型分析。在该研究中,通过将构象可切换的DNA探针与外泌体表面的蛋白标记特异性结合,再利用杂交链反应(HCR),可将单颗粒外泌体的总体尺寸扩大到500 nm以上。然而,该方法的预处理过程较长,且需要设计出大量不同的探针和扩展发夹才能够实现单颗粒外泌体的高通量分析。Liu等^[68]^通过pH介导的组装系统将单个纳米级外泌体转化为微米级的簇,突破了外泌体分析的尺寸限制,使其可以直接用常规的FCM进行分析。通过将pH响应性的二酰基脂质共轭聚合物(DLP)嵌入外泌体的外膜中来控制外泌体的表面润湿性(酸度系数(p*K*_a_)为6.5),在弱酸性缓冲液(pH 5.5)中,外泌体均匀分散在溶液中;在中性缓冲液(pH 7.4)中,DLP变为疏水状态,形成均匀的胶束。此外,该研究发现,黏蛋白1(MUC-1)与程序性死亡配体1(PD-L1)的结合能构成一种新的生物标志物组合,并有助于促进肝癌(HCC)的早期诊断。Tian等^[69]^通过减小探针体积来降低背景噪声,通过延长粒子输运时间来增加光子产生,并结合光子爆发检测技术,研发出了一种高灵敏度流式细胞仪(HSFCM)。该仪器具备三通道同时检测的能力,既可以利用侧散射分析外泌体的大小,也可以通过两种荧光标记来检测外泌体的生物标志物。通过对50 μL血浆样本中的跨膜糖蛋白(CD147)阳性外泌体颗粒含量进行测定,该仪器实现了对结直肠癌的早期诊断及预后分析。

纳米流式检测(nano flow cytometry, nFCM)技术是一种高灵敏度的分析手段,通过检测纳米颗粒的散射光和多色荧光信号,nFCM技术能够在单颗粒水平上对外泌体等纳米尺度颗粒进行多参数定量表征。Cai等^[70]^结合高碘酸盐氧化和苯胺催化肟连接(PAL)反应,并利用实验室自制的nFCM进行单颗粒分析,从而实现对单颗粒外泌体表面唾液酸(SA)的原位标记与分析。结合nFCM技术,该方法能够同时检测单颗粒外泌体的侧向散射光和荧光。Liu等^[71]^同样利用nFCM技术,成功检测到直径低至40 nm的单颗粒外泌体的散射信号,以及经绿色荧光染料(SYTO 16)染色后的单个DNA片段(含200个碱基对)的荧光信号(图3)。这一发现证实了外泌体内部及膜上存在片段化的DNA,且这些DNA主要以双螺旋形式存在。该研究为深入理解DNA与外泌体之间的关联提供了直接且确凿的实验证据。Silva等^[72]^利用nFCM技术对单颗粒外泌体上的绿色荧光蛋白(GFP)信号进行了表征。结果显示,在含有外泌体跨膜蛋白(TSPAN14、CD63和CD81)的样品中,GFP阳性的外泌体比例最高。此外,通过采用单分子定位显微镜(SMLM)技术,从单颗粒和单分子层面对外泌体中蛋白的装载效率进行分析,结果发现,在促进GFP装载至外泌体的过程中,TSPAN14、CD63和CD81均表现出显著效果,其中CD63携带的GFP信号值最强,因此CD63有望成为外泌体载药工程化改造的理想靶点。

**图3:**
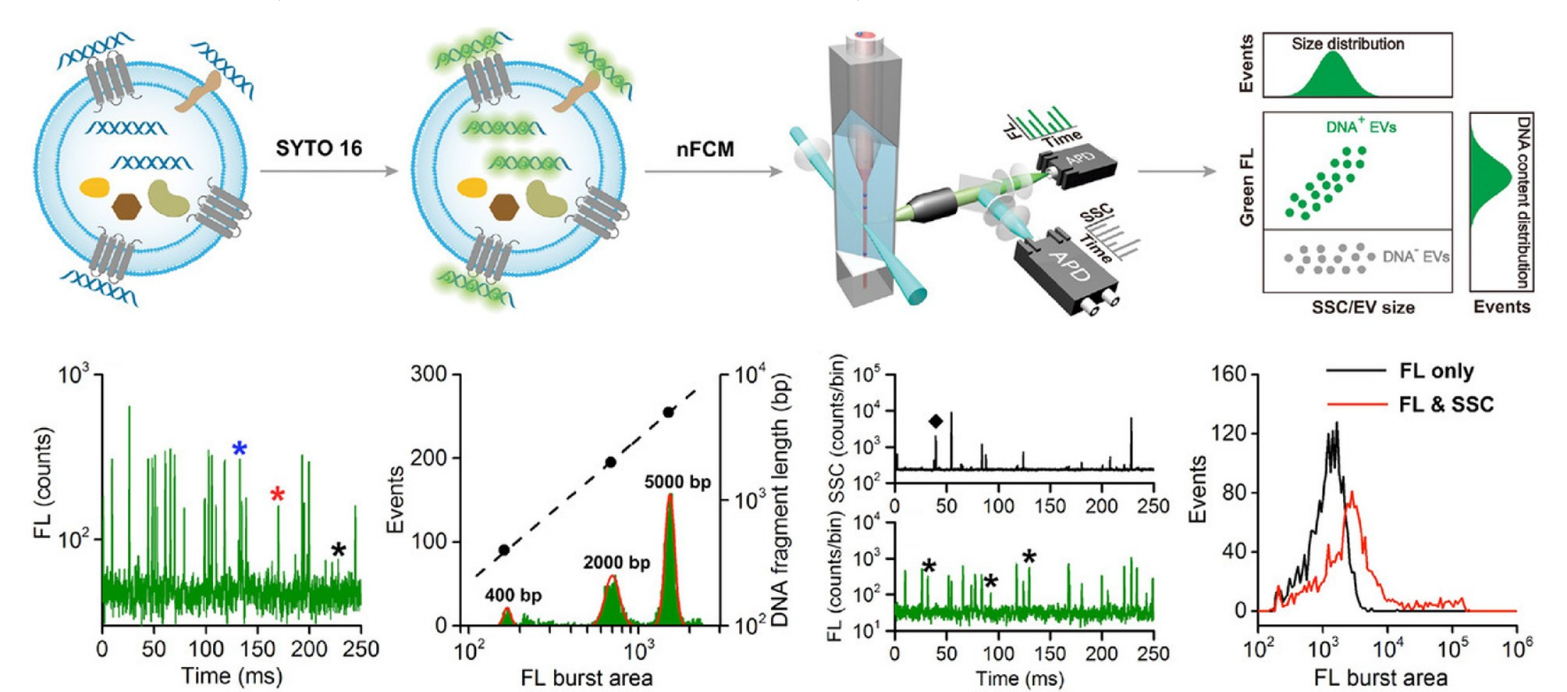
利用nFCM技术在单颗粒水平上进行外泌体DNA分析的示意图^[71]^

成像流式细胞术(imaging fow cytometry, IFCM)是一种结合了流式细胞术与成像技术的方法,它在检测直径超过200 nm的血液颗粒时展现出足够的灵敏度,但其在检测单颗粒外泌体方面的潜力尚需进一步探索。Woud等^[73]^基于IFCM,在不预先分离外泌体的前提下,对缺乏血小板的血浆源性外泌体(直径≤400 nm)进行表型分析和含量测定。研究过程中,采用商用聚苯乙烯珠进行尺寸和荧光仪器校准,并选用羧基荧光素二乙酸琥珀酰亚胺酯(CFSE)、四跨膜蛋白超家族、外泌体跨膜蛋白CD9和CD31作为组合标记物。结果显示,在单颗粒外泌体水平上,超过90%的外泌体展现出双阳性荧光信号。该研究为利用IFCM分析外周血浆中的外泌体提供了参考。Görgens等^[74]^使用结合了增强型绿色荧光蛋白的CD63(CD63eGFP)对外泌体进行标记,并将其作为生物参比材料,在Amnis ImageStreamX MkII成像流式细胞仪上,定义并优化了IFCM的采集与分析参数,实现了对单颗粒外泌体的检测。Ricklefs等^[75]^通过整合多种软件设置并优化IFCM的多个参数,成功实现了来自人和鼠细胞培养物以及血浆样品中外泌体跨膜蛋白CD9、CD63和CD81的分析。研究结果显示,IFCM能够在体外环境中及单颗粒水平上实现对患者血浆中外泌体的多参数表型分析。

### 2.2 超分辨显微镜

超分辨显微镜是对几种空间分辨率超过衍射极限的技术的统称^[76]^。在单颗粒外泌体成像领域中,一个主要研究方向是进行免疫表型分析。尽管外泌体跨膜蛋白CD9、CD63和CD81被广泛用作外泌体的标记物,它们的表达却呈现出高度异质性。Han等^[77]^利用带有多个偶联抗体标记的荧光探针,并结合TIRFM技术,对单颗粒外泌体进行了成像与共定位分析。研究揭示,在由MCF-7细胞分泌的外泌体中,大多数仅含有CD9、CD63和CD81中的一种或两种,而仅有约10%的外泌体同时表达了这3种蛋白;对于小鼠黑色素瘤细胞(B16BL6)分泌的外泌体,只有约1%的外泌体同时表达了这3种蛋白,大多数外泌体只表达了其中一种。使用单一种类的抗体或适配体所捕获到的外泌体是不完整的,有时甚至仅能捕获到外泌体群体中相当小的一部分。为了标记大多数外泌体,需要对多个外泌体标记物进行染色。除了TIRFM外,常用的SRM还包括随机光学重建显微镜(STORM)、单分子定位显微镜(SMLM)、定量单分子定位显微镜(qSMLM)、膨胀显微镜(ExM)等。McNamara等^[78]^利用直接随机光学重建显微镜(dSTORM)技术对单颗粒外泌体的表面微域进行了三维成像,成功识别出单颗粒外泌体以及不同尺寸外泌体表面上的四跨膜蛋白簇。该dSTORM技术在横向(X轴和Y轴)上实现了±16 nm的分辨率,在轴向(Z轴)上达到了±42 nm的分辨率。Saftics等^[79]^利用单细胞外囊泡纳米显微镜(SEVEN),从体积低至0.1 μL的血浆(未经过进一步复杂处理)中检测到了外泌体,并发现了胰腺癌的诊断标志物。SEVEN将免疫亲和捕获技术与定量单分子定位显微镜(qSMLM)技术结合,成功分析了外泌体的数量、大小和形状以及跨膜蛋白的含量和异质性。Wei等^[80]^通过结合超分辨率三色荧光共定位技术(SR-TFC)、三色超分辨率成像技术与计数共定位荧光像素点法(CFPP),开发了一种外泌体蛋白分析策略(图4)。

**图4:**
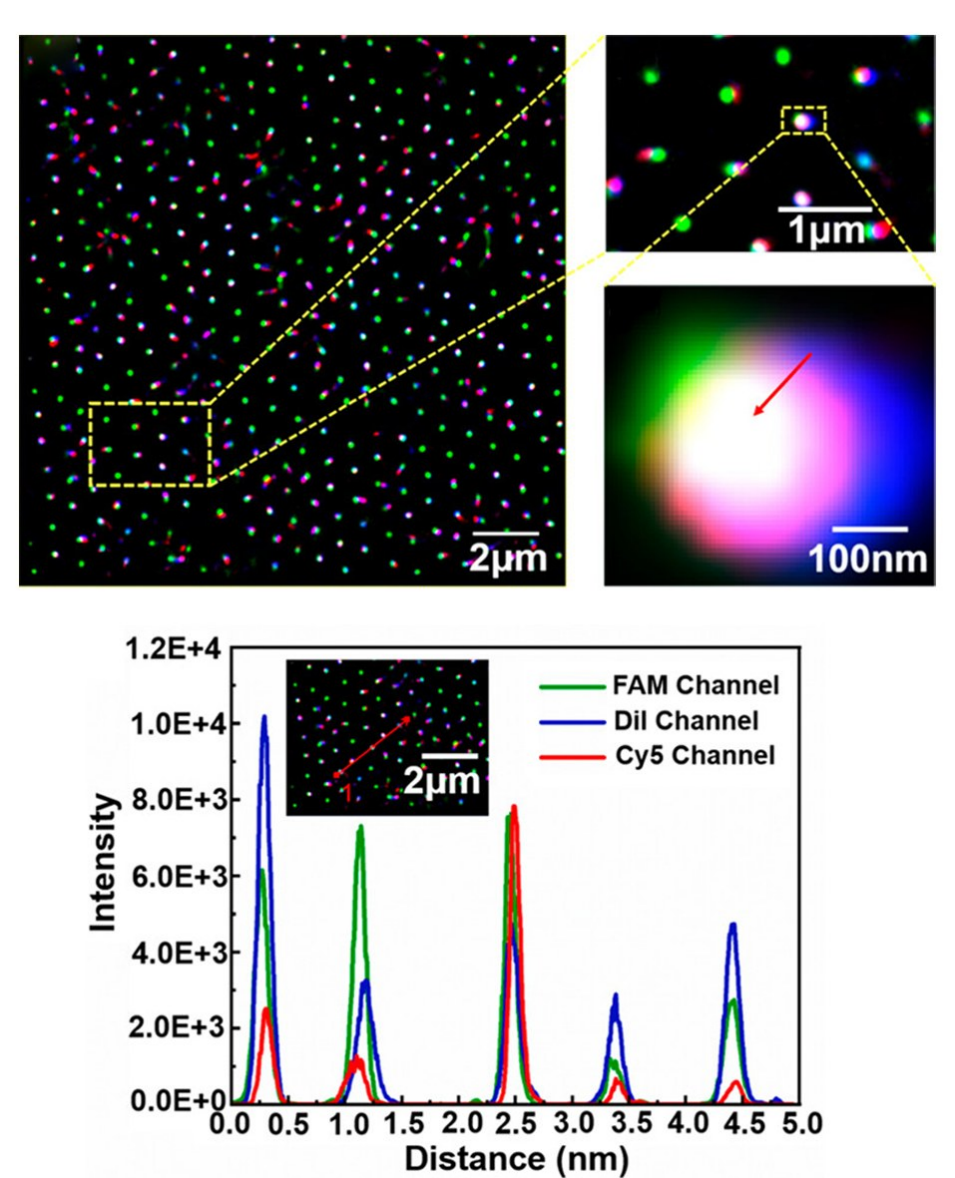
基于SR-TFC、三色超分辨率成像技术与CFPP的外泌体蛋白分析策略^[80]^

该策略能够基于外泌体上的特异性蛋白实现癌症表型的分析和分类。研究中采用经典的三明治免疫测定方案,并利用SMLM技术将捕获探针、外泌体和免疫探针进行共定位。在特异性免疫结合位点处,外泌体、捕获探针及免疫探针的空间位置高度重合,而在非特异性结合位点则不发生重合。ExM技术通过将细胞结构与凝胶中的高分子链进行偶联,并利用凝胶吸水膨胀的过程带动细胞结构的同步膨胀,该技术在普通光学显微镜下即可获得高分辨率的细胞结构成像图。Sun等^[81]^利用生物素点击反应,并结合荧光标记的链霉亲和素进行染色,在ExM体系中成功实现了小分子药物以及脂质、聚糖等生物大分子的高精度成像。基于ExM的高分辨率特性,该技术有望成为研究单颗粒外泌体的有力工具。总之,SRM技术已成为单颗粒外泌体表征的新兴工具,其在单颗粒外泌体研究中的应用范围仍有待进一步拓展。如何将SRM与微流控技术结合,并充分发挥两种技术的优势,对单细胞外泌体或单颗粒外泌体的研究具有重要意义。

### 2.3 原子力显微镜

原子力显微镜通过检测待测样品表面与微型力敏感元件之间的极微弱原子间相互作用力来进行分子表征。与仅能提供二维图像的电子显微镜不同,AFM能够展现外泌体颗粒表面的真实三维形貌及其力学性能。此外,AFM还能够测定外泌体的尺寸分布,并对膜蛋白的组成进行分析。Ye等^[82]^利用AFM上的纳米压痕技术,在单颗粒水平上定量表征了乳腺癌衍生外泌体的纳米力学特性,并探究了这些特性与肿瘤恶性程度之间的关系。AFM上的纳米压痕记录了单颗粒外泌体在局部位置的纳米力学响应,通过分析力压痕曲线(FICs),可以定量提取出与单颗粒外泌体纳米力学特性相关且可量化的具体参数。在尖端纳米压痕过程中,单颗粒外泌体的纳米力学行为能够反映出外泌体在生理和病理状态下对纳米机械刺激的反应特性。LeClaire等^[83]^通过运用多参数AFM技术,成功实现了对乳腺癌细胞及其分泌的单颗粒外泌体在结构和力学性质上的直接对比分析。这一研究结果表明,单颗粒外泌体的生物力学指纹技术作为一种无标记的正交方法,可用于评估亲代细胞的状态。Feng等^[84]^利用AFM技术,在溶液环境中对临床血液癌症患者的液体活检样本中的单颗粒外泌体进行了原位测量,探究了其黏弹特性和几何特征。测量结果揭示了血液癌症发生与发展过程中外泌体力学特性的动态变化。研究结果发现,癌症患者与健康人的外泌体力学特性之间存在显著差异,表明外泌体的力学特性在血液癌症进程中具有指示作用,对推动癌症液体活检技术的研究具有积极意义。Bairamukov等^[85]^利用AFM技术,在空气和液体介质中,对单颗粒水平的血浆外泌体进行了生物力学特性的揭示。综上所述,AFM技术是表征单颗粒外泌体力学性能的主要工具。

### 2.4 SERS

SERS是一种基于等离子体效应的非破坏性、无标记技术。当分析物被置于粗糙的金属表面或纳米颗粒附近时,光激发会引发局部表面等离子体共振,从而显著增强拉曼信号。利用SERS技术对外泌体进行分析时,能够使检测灵敏度提升至单颗粒水平。Li等^[86]^开发了一种单分子分辨率数字外泌体计数检测(DECODE)芯片,该芯片利用金属纳米柱阵列捕获外泌体,并通过SERS技术表征外泌体上生物标志物的表达情况。在一个包含33人(包括有恶性和良性肺结节的患者以及健康个体)的队列中,Li等^[86]^评估了这些标志物在单颗粒外泌体水平上的表达谱,以区分恶性肺结节、良性肺结节或健康对照者,结果显示该方法在肺癌患者的筛查过程中具有很高的可信度。SERS技术能够在不使用任何标签的情况下对目标物进行分析,这一特性能够保证样品的固有特性不受影响。Jalali等^[87]^将MoS_2_等离子体纳米腔与SERS技术结合,在单颗粒水平上对胶质母细胞瘤(GBM)的外泌体进行了研究。研究利用脂质膜与单层MoS_2_之间的相互作用,无需生物识别元件即可在纳米腔中捕获外泌体。这些纳米腔的尺寸足够小,仅能容纳单个粒子,从而确保从单个空腔采集到的拉曼光谱直接对应于单颗粒外泌体(图5)。Lin等^[88]^通过独特的光还原技术将银纳米颗粒嵌入多层黑磷纳米片中,制备了一种具有特殊结构的纳米材料(Ag/BP-NS)。将Ag/BP-NS作为SERS传感器,通过偏振-映射方法实现了对单个罗丹明6G(R6G)分子的可视化SERS检测和拉曼成像;进一步结合机器学习方法,该传感器可以实现对不同肿瘤外泌体的识别。Yan等^[89]^开发了一种芯片,该芯片利用石墨烯覆盖金表面,并设计有周期性的金字塔纳米结构。结合SERS技术,该芯片能够实现单颗粒外泌体的分析。通过对人类血清、细胞培养基及两种肺癌细胞系分泌外泌体的拉曼光谱进行比较,发现这些样本各自展现出独特的拉曼光谱特征。进一步利用主成分分析(PCA)方法,成功地将不同来源的外泌体进行了聚类区分。这一研究为SERS平台在生物医学领域的应用提供了强有力的支持。上述研究结果表明,SERS技术能够用于表征和分析单颗粒外泌体。在未来的研究中,将微流控技术与SERS技术结合,并应用于单颗粒外泌体的表征,有助于更深入地理解外泌体的异质性。

**图5:**
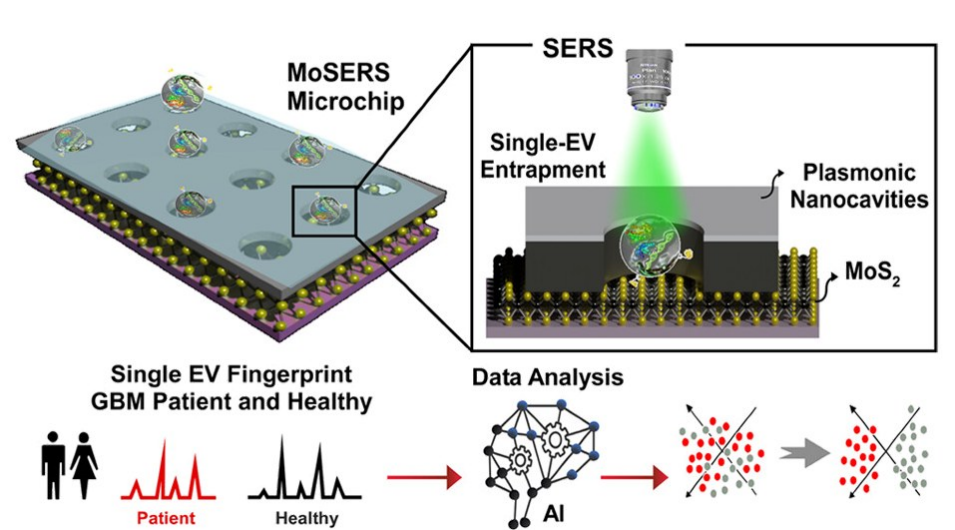
MoS_2_等离子体纳米腔与SERS技术应用于GBM的外泌体分析^[87]^

### 2.5 质谱

质谱是一种兼具高灵敏度、高特异性和高通量等优点的非标记检测技术,能够精确测定蛋白质的表达水平、氨基酸序列及其翻译后修饰。Zhang等^[90]^建立了一种用于外泌体膜识别的适配体-邻近连接激活滚动圈扩增(RCA)方法,并结合单粒子电感耦合等离子体质谱(SP-ICP-MS)技术,实现了对肿瘤外泌体的高灵敏检测及癌症诊断。在该研究中,当DNA标记的超小型金纳米颗粒探针与RCA产生的长链结合后,会聚集形成较大的颗粒。Lee等^[91]^利用纳米弹二次离子质谱(NP-SIMS)技术,实现了对单颗粒外泌体的高灵敏度和多重表征。具体操作流程如下:首先,从肝细胞中分离出外泌体,并利用抗体Abs功能化的Au基底来捕获这些外泌体;接着,使用携带有镧系元素标签(Ln-tags)的抗体对外泌体进行特异性标记;完成标记步骤后,采用NP-SIMS技术对基底表面进行轰击,促使与外泌体结合的Ln-tags得以释放。该方法能够高效地检测单颗粒外泌体表面的标记物,为深入探究外泌体的特性提供了强有力的工具。质谱流式细胞术(mass cytometry, CyTOF)融合了传统流式细胞术和MS技术。CyTOF使用高稳定性金属元素(主要是镧系元素及其同位素)标记的抗体来特异性识别并结合目标抗原。这些标记了抗体的金属离子在离子化后,以离子流的形式传输至飞行时间质量分析器,最终产生独特的金属离子信号峰(ICP质谱成像)。Wang等^[92]^通过CyTOF将金属标签标记在外泌体上,从而实现了体内单细胞水平的外泌体研究。通过电穿孔技术,将含金属同位素的嵌入剂装载到外泌体中,随后利用细胞计数技术在单细胞水平上追踪这些标记的外泌体,并在乳腺癌小鼠模型中验证了这些外泌体的抗癌效果。

### 2.6 邻近编码技术

邻近编码技术是一种创新的单颗粒外泌体表面蛋白分析技术。该技术利用抗体-DNA偶联物来精确识别并结合外泌体的表面蛋白。同时,通过RCA生成的DNA序列对单颗粒外泌体进行分隔与独特标记。随后,采用高通量测序技术对这些DNA序列进行分析,从中提取关于蛋白质和外泌体的关键信息,最终成功获取单颗粒外泌体的蛋白质组学数据^[93]^。Guo等^[94]^将PBA用于结直肠癌患者血浆中混杂外泌体的解析,实现了对样本中数十万个外泌体上多达200种膜蛋白的定量检测。研究结果显示,随着结直肠癌的发生、发展和转移,患者血浆中的外泌体亚群构成呈现出规律性的变化,其中整合素αM(ITGAM)阳性外泌体亚群对结直肠癌具有抑制作用,整合素β3(ITGB3)阳性外泌体亚群具有促癌作用。Cai等^[95]^基于PBA,在单颗粒水平上,对阿尔茨海默病(AD)患者及AD小鼠模型的5种体液(鼻灌洗液、口腔灌洗液、眼灌洗液、尿液和血液)中的外泌体表面蛋白进行了多重分析,以评估这些体液作为AD生物标志物来源的潜力。实验结果表明,不同体液中的外泌体表面蛋白谱存在显著差异,其中尿液中的外泌体是更好的AD诊断标志物。Min等^[96]^采用PBA对食管癌、胃癌、结直肠癌、肝癌和肺癌患者(共100名)以及100名健康对照者血浆中的外泌体表面蛋白进行了分析。基于这些样本中鉴定出的差异表达蛋白(DEPs)及其组合(DEPCs),构建了一个分类模型,该模型在泛癌诊断中表现出了优异的性能,为外泌体蛋白在癌症预测领域的临床应用提供了有力依据。Sun等^[97]^利用PBA在单颗粒外泌体水平上进行蛋白质组解析,对人体中的100种蛋白质及严重急性呼吸综合征冠状病毒2(SARS-CoV-2)核衣壳蛋白进行了联合检测。通过该研究,他们发现了与病毒共定位的外泌体亚群,明确了这些外泌体亚群的蛋白图谱特征,并揭示了这些特征在病毒感染过程中的动态变化规律。综上所述,PBA是一种成熟的单颗粒外泌体表征与分析技术,在临床应用中具有广泛的适用性。

Olink蛋白组学基于邻近延伸分析(proximity extension assay, PEA)技术来实现对样本中蛋白质的高通量检测。该技术的原理在于,使用一对特异性抗体来识别并结合靶蛋白,这对抗体各自偶联有DNA寡核苷酸标记。当结合在同一靶蛋白上的抗体相互靠近时,抗体所携带的DNA寡核苷酸会因空间邻近而互补配对,进而在DNA聚合酶的作用下进行延伸。通过后续的实时荧光定量聚合酶链反应(qPCR)或二代测序技术,可以对这些DNA寡核苷酸进行定量分析,而特定的核苷酸序列信号则反映了相应蛋白质的含量^[98]^。Bryl-Górecka等^[99]^为提高血浆蛋白质检测的灵敏度,使用Olink蛋白组学技术表征和分离外泌体,首次报道了运动前后人体中外泌体蛋白质差异表达的数据。结果显示,运动后外泌体中有54种蛋白质发生显著变化,而不含外泌体的血浆蛋白质组中仅有4种蛋白质的表达出现差异调节,这凸显了这些外泌体在细胞通讯中的重要作用以及它们作为血浆衍生生物标志物的潜力。Bedin等^[100]^利用Olink蛋白组学技术,通过对两种单克隆人血浆降钙素原(PCT)抗体进行直接捕获和检测,在较宽的线性范围内实现了PCT的高特异性和高灵敏度检测;与使用PEA所得到的结果相比,Olink蛋白组学技术将测定时间缩短13倍以上,且检出限低至0.1 ng/mL。Olink蛋白组学技术在原理上与PBA有很大的相似性,但它尚未被应用于单细胞和单颗粒外泌体的检测。鉴于Olink蛋白组学技术在血浆蛋白质检测中展现出的高灵敏度,它为单细胞和单颗粒外泌体的分析提供了新的可能性。

### 2.7 其他技术

除了上述几种常见的单颗粒外泌体表征技术之外,还有几种较为适用的技术,包括表面等离子共振、暗场显微镜成像、电化学传感技术以及纳米红外光谱(nano-FTIR)技术等。Zhai等^[101]^结合表面等离子体共振显微镜(SPRM)与单颗粒外泌体表面蛋白谱动态免疫分析(DISEP)方法,对血浆中的肿瘤源性单个外泌体进行数字化定量,并实现了高特异性的表面蛋白谱分析。Liang等^[102]^报道了一种纳米等离子体增强散射(nPES)测定方法,该方法结合暗场显微镜技术,能够直接从1 μL的血浆样本中定量检测肿瘤来源的外泌体。Zhang等^[103]^开发了一种局部荧光成像(DPPIE)方法,用于分析单个外泌体上的多种蛋白质。研究使用CD9作为捕获抗体,将捕获到的外泌体固定在芯片表面;随后,这些外泌体会与含有滚环扩增序列的CD63、EpCAM、MUC1适配体特异性结合,并产生局部荧光信号。Lv等^[104]^构建了一种基于金膜沉积的玻璃管纳米电极传感策略,用于检测单个外泌体及其负载的多巴胺(DA)含量。通过在玻璃纳米管电极尖端内壁喷涂金纳米颗粒,制备金膜沉积的纳米电极,在恒定电位下,整个金膜区域会形成等电位。该纳米电极能够监测单个外泌体通过时的信号,并通过测量电流-时间曲线来检测单个外泌体中的DA含量。该研究为电化学生物传感器的设计提供了新的思路。Xue等^[105]^运用nano-FTIR技术,探究了单颗粒外泌体中与恶性肿瘤相关的蛋白质的异质性。他们对比了源自不同人乳腺癌细胞系以及来自有或无转移的乳腺癌患者原发肿瘤组织的外泌体蛋白质的nano-FTIR图谱。研究结果显示,外泌体的nano-FTIR特征在评估肿瘤恶性程度和预测转移情况方面展现出了较高的灵敏度和特异性。然而,单颗粒外泌体的红外散射信号较弱,且nano-FTIR技术在筛选和采集不同来源(如不同恶性程度的人乳腺癌细胞系、乳腺癌患者的原发肿瘤组织等)的外泌体样品数据时耗时较长。该方法在大规模临床分析领域,尤其是针对癌症衍生外泌体含量有限的早期癌症检测方面,面临着挑战。因此,开发一种能够快速且大规模表征外泌体的nano-FTIR方法,对于推动其在临床实际应用中的进展至关重要。Schürz等^[106]^开发了一个可从成像数据中自动定量分析单个颗粒的ImageJ插件(EVAnalyzer)。EVAnalyzer基于标准共聚焦和广角荧光显微镜衍射极限限制下的光斑检测原理,通过采用特定的标准试剂、材料和设备来实现外泌体的捕获、免疫标记及荧光成像。这一创新技术显著降低了实验成本,为单个外泌体的研究带来了极大的便利。

点击化学和分子印迹技术在单细胞及单颗粒外泌体分析方面具有极大的潜力。Ma等^[107]^利用Cu触发的叠氮修饰CD63适配体与炔基功能化聚合物点(alkyne-pdots)之间的点击反应,创新性地开发了一种荧光检测方法,该方法专门用于乳腺癌外泌体的检测。Cao等^[108]^基于跨膜糖蛋白(CD44+)外泌体模型的接近标记辅助点击共轭方法,成功地实现了循环外泌体中特定亚群的电化学分析。Zhou等^[109]^开发了一种基于反相微乳液体系的磷脂分子印迹方法。该方法基于两亲性磷脂分子在油-水界面上的有序自组装排列,构建了针对磷脂亲水表位基团的分子印迹材料,并实现了对细胞质膜上磷脂双分子层的有效识别;同时,该分子印迹材料还能够对磷脂特异性表达的外泌体进行选择性富集。Xie等^[110]^利用易获取的结构类似物(CoA)作为虚拟模板,通过反向微乳液模板对接表面印迹方法,制备了能够特异性识别4'-磷酸泛酰巯基乙胺化(4PPTylation)的拉曼纳米标签,为单细胞分辨率的分析提供了一种有力的工具。未来需探索如何将这些技术与微流控平台结合,以实现单细胞的培养与外泌体的分离,进而开展单细胞与单颗粒外泌体的一体化研究。

## 3 总结与展望

目前,单细胞和单颗粒外泌体的研究仍处于初级阶段,相关研究方法和技术正迅速发展。微流控技术在单细胞外泌体分析领域展现出了巨大的发展潜力。本文综述了微流控技术在单细胞分离培养及外泌体分离分析方面的最新进展,并系统归纳了常用的单颗粒外泌体表征技术,包括FCM、SRM、AFM、SERS、MS和PBA等,旨在为初学者在微流控平台设计方面提供指导,并帮助他们找到最适合的单细胞和单颗粒外泌体研究方法。未来,随着Olink蛋白组学、点击化学和分子印迹等技术在单细胞及单颗粒外泌体分析领域的不断进步,有望取得一系列重大突破。一方面,这些技术与微流控平台的深度融合不仅限于实现单细胞的培养与外泌体的分离,还能在芯片上集成多种功能模块。例如,构建集细胞培养、外泌体实时监测、原位分析及分子标记等功能于一体的微流控系统,这将显著提升分析通量和效率,实现对单细胞及单颗粒外泌体的高通量、高灵敏度及高特异性分析。另一方面,随着人工智能和大数据技术的蓬勃发展,将它们与这些新兴的外泌体分析技术相结合将成为一个极具潜力的研究方向。利用人工智能算法对大量单细胞及单颗粒外泌体数据进行深度挖掘和分析,可以自动识别和分类外泌体特征,从而建立更为精准的癌症诊断模型。同时,大数据分析能够整合不同来源、不同类型的数据,为揭示外泌体在癌症发生发展过程中的复杂机制提供强有力的支持。综上所述,Olink蛋白组学、点击化学和分子印迹等技术与微流控平台、人工智能、大数据等技术的融合,将为单细胞及单颗粒外泌体的分离分析带来广阔的发展前景,为生命科学研究和临床应用开辟新的道路。

## 作者团队简介

生物识别材料与蛋白质组学课题组隶属于吉林大学超分子化学生物学研究中心,研究组致力于生物分子识别与蛋白质组学新方法的开发及其在生物医学研究中的应用。近年来,研究团队发展了针对外泌体、细菌与病毒等生物体系的分子印迹聚合物和超分子探针等分子识别工具,可对各种临床体液样本和单细胞的外泌体进行高通量富集与多组学分析,为疾病标志物的发现和肿瘤异质性研究提供新的策略和方法。

课题组微信公众号:Biorecognition and Proteomics

**Table:** 

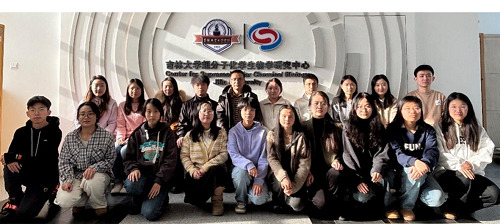	人才队伍
	**课题组组长:**胡良海教授 **课题组成员及学生:**教授1人,“鼎新学者”博士后、研究生及本科生20余人 **团队精神:**交叉融合,协作共进,以色谱之工具,探生命之奥秘

**Table:** 

科研项目及成果	研究领域
**科研项目:**国家自然科学基金,人体蛋白质组导航国际大科学计划(π-HuB计划)“种子项目”和吉林省科技厅基金等 **科研成果:**开发了基于分子印迹原理的细胞、细菌、病毒和外泌体识别新方法,为肿瘤的靶向和感染性疾病的诊疗提供了新策略;发展了基于超分子探针的外泌体阵列捕获和原位蛋白质分析的一体化平台,将有力推动疾病标志物的临床大队列研究。在*Nat Protoc*、*Angew Chem Int Ed*、*Anal Chem*、*J Chromatogr A*和《色谱》等期刊上发表论文90余篇,申请国家专利10余项,PCT专利1项。 **获奖情况:**入选教育部“新世纪优秀人才”支持计划,担任中国质谱学会和蛋白质组学会学术委员,担任*Chinese Chemical Letters*和《色谱》期刊青年编委;获中国科学院院长优秀奖,中国分析测试协会科学技术奖(CAIA奖)青年奖,吉林省自然科学学术成果二等奖等;团队成员荣获全国色谱会优秀青年报告奖、HPLC Tube视频奖和优秀墙报奖等学术奖励。